# The impact of comorbidities and economic inequality on COVID-19 mortality in Mexico: a machine learning approach

**DOI:** 10.3389/fdata.2024.1298029

**Published:** 2024-03-18

**Authors:** Jorge Méndez-Astudillo

**Affiliations:** Institute of Economic Research, National Autonomous University of Mexico, Mexico City, Mexico

**Keywords:** Random Forest feature importance, XGBoost feature importance, comorbidities, deprivation, public health, COVID-19

## Abstract

**Introduction:**

Studies from different parts of the world have shown that some comorbidities are associated with fatal cases of COVID-19. However, the prevalence rates of comorbidities are different around the world, therefore, their contribution to COVID-19 mortality is different. Socioeconomic factors may influence the prevalence of comorbidities; therefore, they may also influence COVID-19 mortality.

**Methods:**

This study conducted feature analysis using two supervised machine learning classification algorithms, Random Forest and XGBoost, to examine the comorbidities and level of economic inequalities associated with fatal cases of COVID-19 in Mexico. The dataset used was collected by the National Epidemiology Center from February 2020 to November 2022, and includes more than 20 million observations and 40 variables describing the characteristics of the individuals who underwent COVID-19 testing or treatment. In addition, socioeconomic inequalities were measured using the normalized marginalization index calculated by the National Population Council and the deprivation index calculated by NASA.

**Results:**

The analysis shows that diabetes and hypertension were the main comorbidities defining the mortality of COVID-19, furthermore, socioeconomic inequalities were also important characteristics defining the mortality. Similar features were found with Random Forest and XGBoost.

**Discussion:**

It is imperative to implement programs aimed at reducing inequalities as well as preventable comorbidities to make the population more resilient to future pandemics. The results apply to regions or countries with similar levels of inequality or comorbidity prevalence.

## Introduction

On March 11th, 2020, the World Health Organization (WHO) declared the new SARS-CoV-2 coronavirus disease a pandemic (WHO, 2020). At the start of the pandemic, there was limited understanding of the dynamics of the virus and the long- and short-term effects it would have on human health (Fauci et al., 2020). As the pandemic spread around the world, it overwhelmed health systems (Sagan et al., 2021) and caused a global excess of deaths (WHO, 2022). As a result, public health authorities took a variety of measures to stop the spread of the virus. This included restricting mobility, stopping mass gatherings, and even halting non-essential economic activities, which had a negative impact on the global economy (World Bank, 2022).

According to the World Development Bank, the economic hardship resulting from the COVID-19 pandemic hit economies with higher inequality harder, especially developing economies (World Bank, 2022). Hospitalizations and vaccination programs increased public spending and the halt in economic activity caused unemployment, which contributed to rising inequality and poverty, especially in developing countries (Lustig and Martínez Pabón, 2021).

Globally, the pandemic caused an excess of deaths (WHO, 2022), but the number of deaths in each country varied for several reasons among them because of comorbidities. Mathematical models have shown that comorbidities contribute to the spread and the severity of the pandemic (Das et al., 2021a,b). Previous studies have associated comorbidities, such as hypertension with severe cases and death from COVID-19 (Baradaran et al., 2020). Furthermore, hypertension, heart disease and diabetes have been associated with a higher probability of a patient's need for intensive care or death from COVID-19 infection (Espinosa et al., 2020). Recent studies have shown that the risk of dying from COVID-19 infection is highest in people with complicated diabetes, obesity, and anxiety-related disorders (Adab et al., 2022). Other comorbidities such as chronic kidney disease, diabetes, lung and liver disease, cardiovascular disease, obesity, and even mental illness increase the risk of death or serious illness (Adab et al., 2022).

Some of the comorbidities associated with severe disease are related to socioeconomic conditions (Garin et al., 2016), such as poor diet or lifestyle. For example, diabetes is associated with both factors, and it has different prevalence rates in different countries (Saeedi et al., 2019). Therefore, different prevalence rates of comorbidities in different countries had different effects on the COVID-19 pandemic. For example, in Saudi Arabia, COVID-19 mortality was associated with diabetes, hypertension, and chronic cardiovascular disease (Al-Otaiby et al., 2022). Data from Côte d'Ivoire showed that diabetes, hypertension, and obesity were the major comorbidities of COVID-19 deaths in that country (Usui et al., 2022).

Social and economic inequalities appear to have a significant impact on the outcome of the COVID-19 infection. Studies using data from the early days of the pandemic, have correlated socioeconomic indicators such as income, poverty, and unemployment with risk of hospitalization and incidence in countries with low economic inequality (Wachtler et al., 2020). According to the Organization for Economic Cooperation and Development (OECD) and the World Bank, Mexico is a country with high levels of inequality (OECD, 2021). Therefore, data from Mexico can be used to better understand the effect of inequality and comorbidities on the outcome of the COVID-19 infection in countries with high income inequality and marginalization. Previous studies have also been shown that social vulnerability is associated with COVID-19 mortality (Johnson et al., 2021) and social vulnerability is associated with marginalization and economic inequality.

Mexican data from the beginning of the pandemic (March and April 2020) showed that people with 2 or more comorbidities were more likely to die from COVID-19 infection (Kammar-García et al., 2020). They also found that diabetes, hypertension, and obesity were significant factors in COVID-19 deaths. In contrast, using data from March to July 2020 (Calixto-Calderón et al., 2021), found that chronic obstructive pulmonary disease was a significant comorbidity in fatal COVID-19 cases. These studies did not consider the economic conditions of the Mexican population. Therefore, this study contributes to the evaluation of the impact of comorbidities and economic inequalities at the same time on COVID-19 mortality.

In Mexico, the National Center for Epidemiology has been collecting data on comorbidities, age, sex, and outcome of the infection for all COVID-19 cases registered by the health authorities and laboratories performing COVID-19 testing since the beginning of the pandemic. In this paper, a machine learning classifier is implemented to investigate the importance of comorbidities and economic inequality in COVID-19 infection deaths through a feature importance study. The dataset used contains ~20 million observations at the national level from February 2020 to November 2022. In addition, the marginalization index of the National Population Council (CONAPO, 2023) and economic deprivation data from the Global Gridded Relative Deprivation Index (University, 2022) were used to measure inequality in Mexico. This study represents the first comprehensive analysis of the pandemic's evolution in Mexico, based on a large dataset containing comorbidity incidence, socioeconomic characteristics of the patients and outcome of the infection.

Due to the amount of data available, machine learning (ML) approaches with the merged datasets are appropriate for the purpose of the study. The feature importance of all comorbidities reported in the dataset and the measure of inequality are obtained from a Random Forest (RF) and XGBoost classifier trained on all available data. This study contributes to the understanding of which comorbidities contribute to COVID-19 mortality in Mexico and how inequality contributes to certain comorbidities and to the outcome of COVID-19 infection. The results contribute to understanding the interventions and public policies needed to reduce comorbidities associated with lifestyle or economic inequalities, thus, making the population more resilient to future pandemics. Additionally, the results suggest that economic marginalization and deprivation must be addressed to enhance the population's resilience to similar pandemics. The conclusions of this study are applicable to other populations with comparable comorbidity rates and economic inequalities.

## Methods and materials

### COVID-19 dataset

The National Center for Epidemiology (DGE in Spanish) has been collecting data on the characteristics of patients being diagnosed or treated for COVID-19 since the beginning of the pandemic in January 2020. The dataset includes 40 variables, such as age, nationality, sex, place of residence, diagnosis, and prevalence of comorbidities for each test performed in Mexico or for each patient admitted to the health system in both, public and private institutions with COVID-19 symptoms. The dataset is updated twice a month, and it is freely available on the website of the National Center for Epidemiology (DGE, 2022). For this study, the November 2022 update was collected and analyzed. It contains 20 million rows of data from January 2020 to October 2022. A total of 8,713,665 patients with a positive diagnosis of COVID-19 by association, test, or medical in the whole country were selected. For each positive COVID-19 infection in the subset, a binary variable was created indicating whether the patient died from COVID-19. A total of 347,965 COVID-19 deaths (3.99% of all reported cases) were reported in the dataset.

### Social and economic inequality in Mexico

The National Population Council (CONAPO) defines marginalization as a structural phenomenon that originates, on the one hand, in the difficulty of diffusing technological progress and the productive structure in certain regions of the country. On the other hand, it results from the exclusion of certain social groups from access to economic development (CONAPO, 2023). CONAPO provides the Index of Marginalization for 2020 based on census data on educational level, housing characteristics, population density and income (CONAPO, 2023). Values of the normalized index closer to 1 indicate lower marginalization or less inequality, and values closer to 0 indicate more marginalization or more inequality.

The Center for International Earth Science Information Network (CIESIN) provides the Global Gridded Relative Deprivation Index (GRDI) to characterize relative levels of multidimensional deprivation at 1 km resolution in a raster format (University, 2022), where a value of 100 represents the highest level of deprivation and a value of 0 represents the lowest. The latest version of the GRDI was calculated using data from 2010 to 2020.

The COVID-19 dataset includes the municipality of residence for each patient. As a result, the marginalization and deprivation indices were calculated at the municipality level. However, this approach has a limitation in that it does not account for variations in marginalization and deprivation within a municipality.

### Random forest

Random Forest (RF) is a supervised machine learning (ML) algorithm based on decision trees (DT) used for classification and regression problems. A DT is an algorithm that allows an input to be based on different levels of nodes. The output of a DT classifier is the class to which the input belongs. A “Random Forest” is formed with an ensemble of individual decision trees (DTs) that are randomly formed from different features involved in the classification. In the RF algorithm, each DT predicts a class, and the class with the most votes becomes the predicted class and the output of the algorithm. The mathematical details of RF are described in detail in Breiman (2001). The RF algorithm has embedded feature importance calculations, so it is useful to study feature importance. The main advantage of the RF algorithm is that it is easy to train with high efficiency, but the graphical visualization of the results can be difficult to understand.

The input to the RF algorithm included a dataset with the following variables.

Age, sex, indication of whether the patient had: Diabetes, Chronic Obstructive Pulmonary Disease (COPD), Asthma, High Blood Pressure (HBP), Cardiovascular Disease, Obesity, Chronic Kidney Disease (CKD), and whether the patient smoked. In addition, the deprivation and marginalization index of the municipality where the patient resides were also included in the dataset for this study. The RF was trained to predict the patient's death. The dataset included an indicator if the patient was diagnosed with the infection and died from the infection. The following parameters were used to train the RF model: bootstrap, maximum depth = 6, function to measure quality of split = gini, and number of trees in the forest = 20.

### XGBoost

Extreme Gradient Boosting (XGBoost) is a supervised ML algorithm based on the RF algorithm. The XGBoost algorithm generates a sequence of DT ensembles, which are sequentially aggregated to improve precision and reduce prediction error. Gradient decent is applied to improve the precision of the algorithm (Chen and Guestrin, 2016). The main advantage of XGBoost is that it can handle multiple variables simultaneously, and it works with high precision. However, it consumes a lot of computational resources, especially for large datasets. Both, RF and XGBoost are supervised machine learning algorithms, which require the dataset to be divided into a training set and a test set.

XGBoost used the same inputs as RF. In both algorithms, 70% of data was used for training and 30% for testing. A learning rate of 0.3, 20 estimators and maximum depth of 6 were used to train the model.

**Figure 1:**
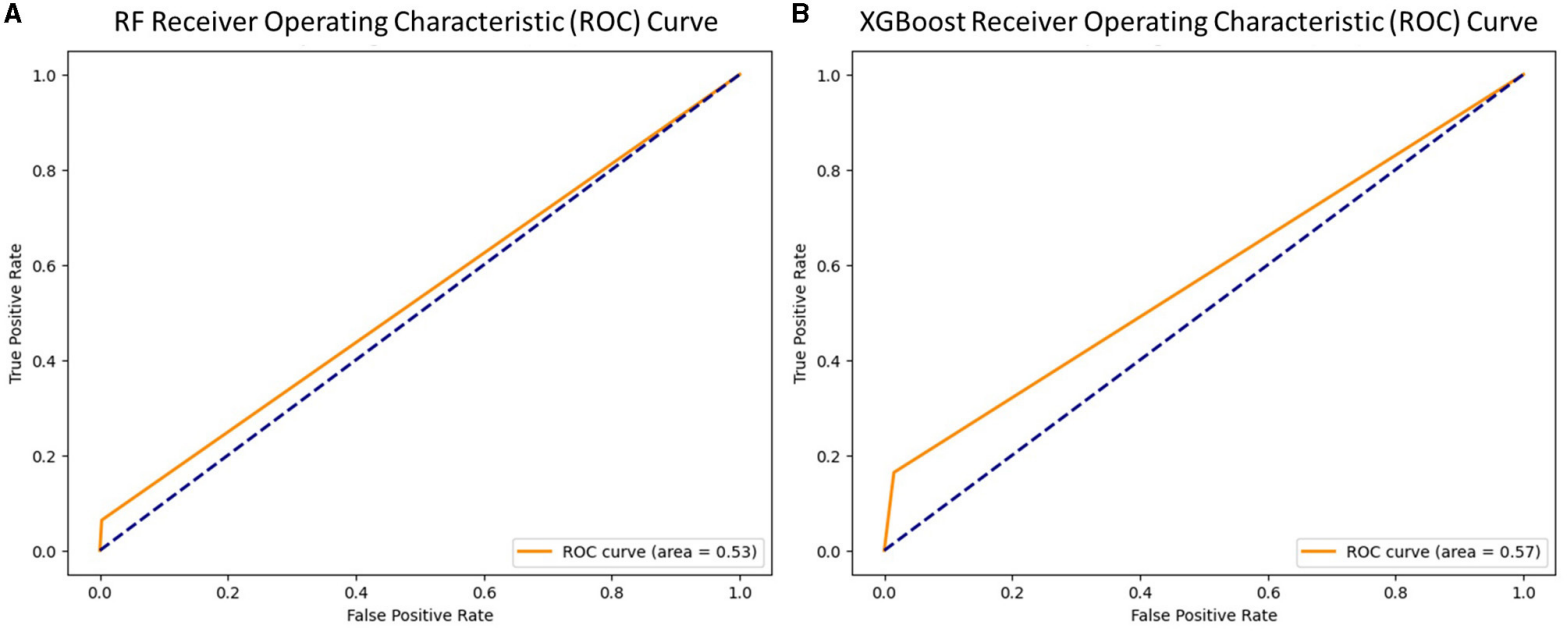
ROC curve for **(A)** Random Forest and **(B)** XGBoost.

The Receiver Operating Characteristic (ROC) Curves for both classifiers are shown in Figure 1 for validation purposes.

Figure 1 shows that XGBoost had a better overall performance than RF for this classification task.

## Results

**Figure 2:**
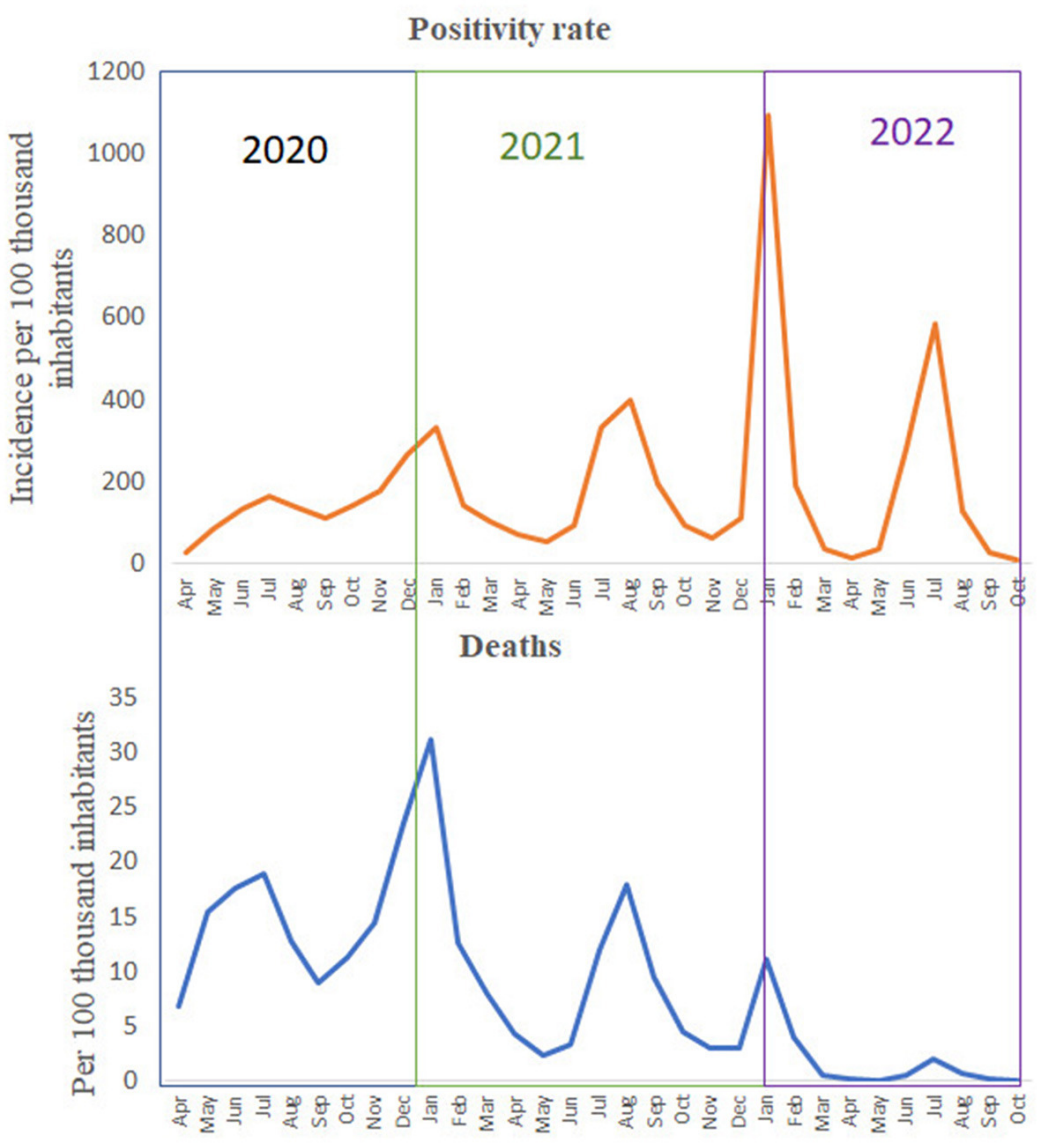
Positivity and death rate per 100,000 inhabitants in Mexico. Data from April 2020 to October 2022.

The dynamics of the pandemic from March 2020 to October 2022 are shown in Figure 2. The positivity rate was defined as the incidence of COVID-19 infection per 100,000 people. Similarly pandemic mortality was defined as the number of deaths attributed to COVID-19 infection per 100,000 inhabitants. Figure 1 shows both, the positivity rate, and the death rate per 100,000 inhabitants in Mexico from March 2020 to October 2022.

As shown in Figure 2, the number of cases and deaths increased from April to December 2020, and both rates reached their peak in December 2020, which was the first peak of the original COVID-19 variant. Then, in 2021, new variants appeared, and both rates increased again, reaching their peak in August 2021. After this peak, both rates decreased until the Omicron variant appeared and peaked positivity and death rates in January 2022. The last peak reported the highest number of infections ever, but the death rate was not as high as in previous peaks.

From March 2022, the death rate per 100,000 inhabitants seems to be constant and very low, possibly due to the effect of the pandemic control measures (such as social distancing, mobility restrictions, and mask wearing mandates) and vaccination programs enacted to control the pandemic. In addition, the last peak of infection in July 2022 did not result in a high mortality rate. Therefore, there was no need to implement new measures and the health authorities' plan called “Living with COVID-19” could be implemented.

## Positivity and fatal cases by sex and age

**Figure 3:**
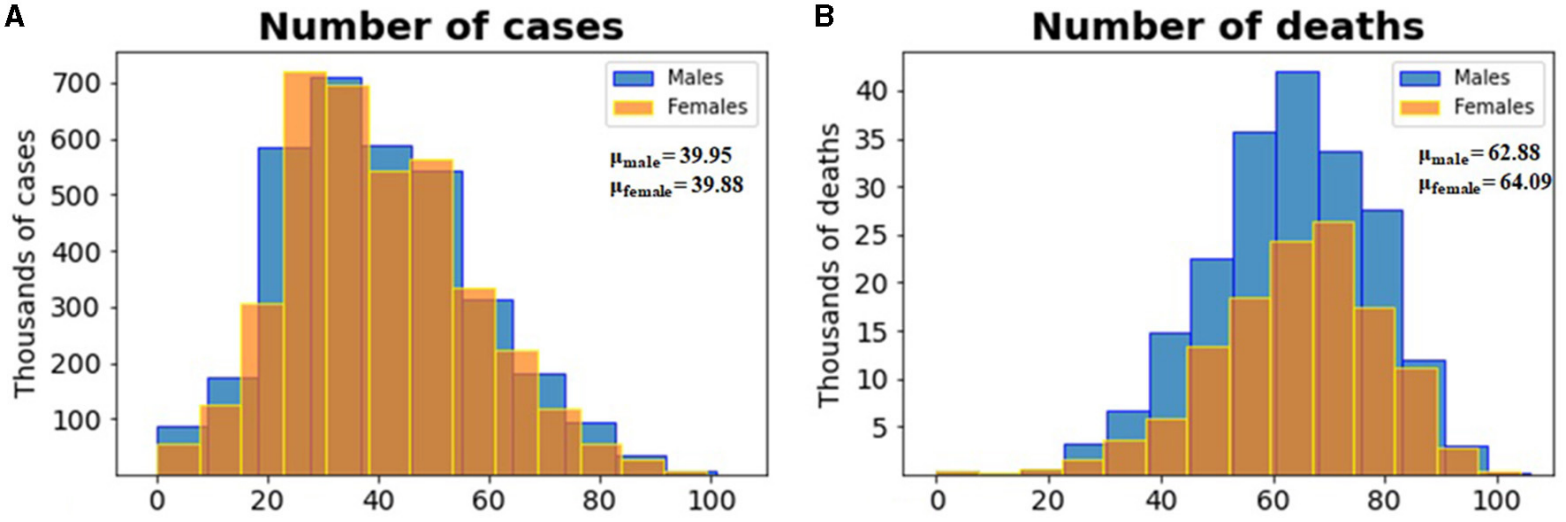
**(A)** Histogram of the distribution of the number of cases per age for both sexes and **(B)** histogram of the distribution of the number of deaths per age for both sexes. In both cases, males are shown in blue and females in yellow.

Figures 3A, B shows the age distribution of confirmed cases and deaths by sex.

According to the histograms in Figure 3A, males and females in the ages around the age of 40 were the most infected. Furthermore, the age distribution is very similar for males and females. The fact that most infections were detected in this age group can be explained by the fact that this age group is very active economically and socially, which requires them to be in contact with many people on a daily basis.

According to the histograms in Figure 3, men and women aged 60 years and older reported few cases but reported a high number of deaths. Therefore, age seems to be an important factor in defining deaths by COVID-19. According to the histograms in Figure 3B, a greater number of deaths were reported by men aged around 62 years and women aged around 64 years. In general, men reported more deaths than women. Therefore, age and sex appear to be important characteristics in the outcome of COVID-19 infection. The fact that most deaths were reported in older patients may be explained by comorbidities and deteriorating of health in this age group. Furthermore, the low infection rate in this age group may be explained by their lower economic and social activity.

**Figure 4:**
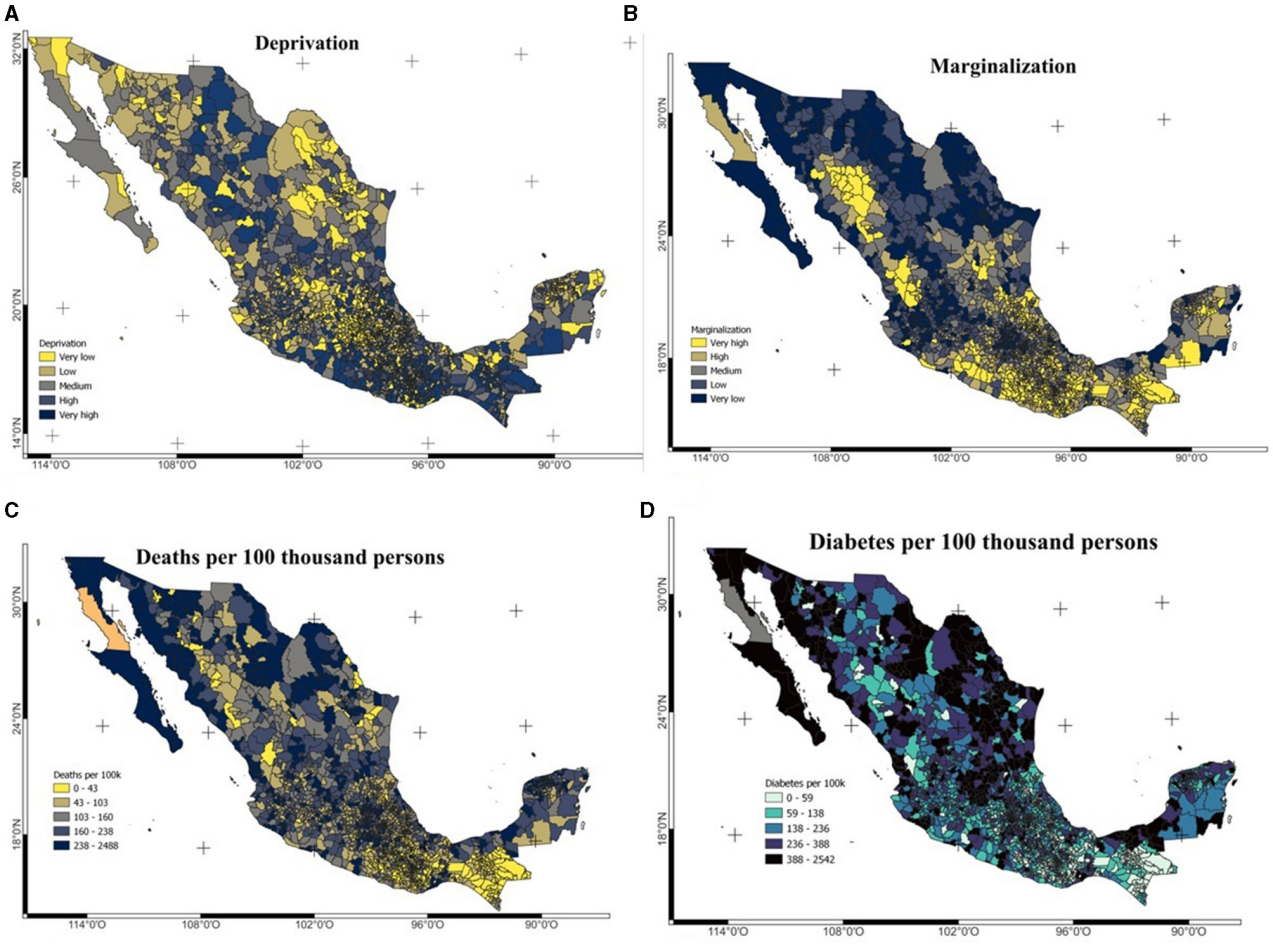
**(A)** Spatial distribution of deprivation, **(B)** marginalization, **(C)** deaths, and **(D)** diabetes prevalence per 100,000 persons. Deaths and diabetes prevalence were calculated from the dataset.

The spatial distribution of deaths and comorbidities per 100,000 people in all municipalities (lowest administrative level) reported in the dataset is shown in the maps in Figure 4. The comorbidities reported in the dataset are diabetes, asthma, chronic obstructive pulmonary disease (COPD), high blood pressure (HBP), cardiovascular disease, obesity, chronic kidney disease (CKD) and smoking. All of these conditions are self-reported by the patients, who indicate whether they have it or not.

Figure 4C shows the average deprivation index (GRDI levels) at the municipal level for all municipalities in Mexico and Figure 4D shows the spatial distribution of economic marginalization at the municipal level as indicated by the CONAPO marginalization index. The main urban areas of Mexico City, Guadalajara, Monterrey, and Merida are those with the lowest levels of marginalization and deprivation. In all cases data at the municipal level was used as the residence of the patients was reported at this level.

## Feature importance with ML algorithms

**Figure 5:**
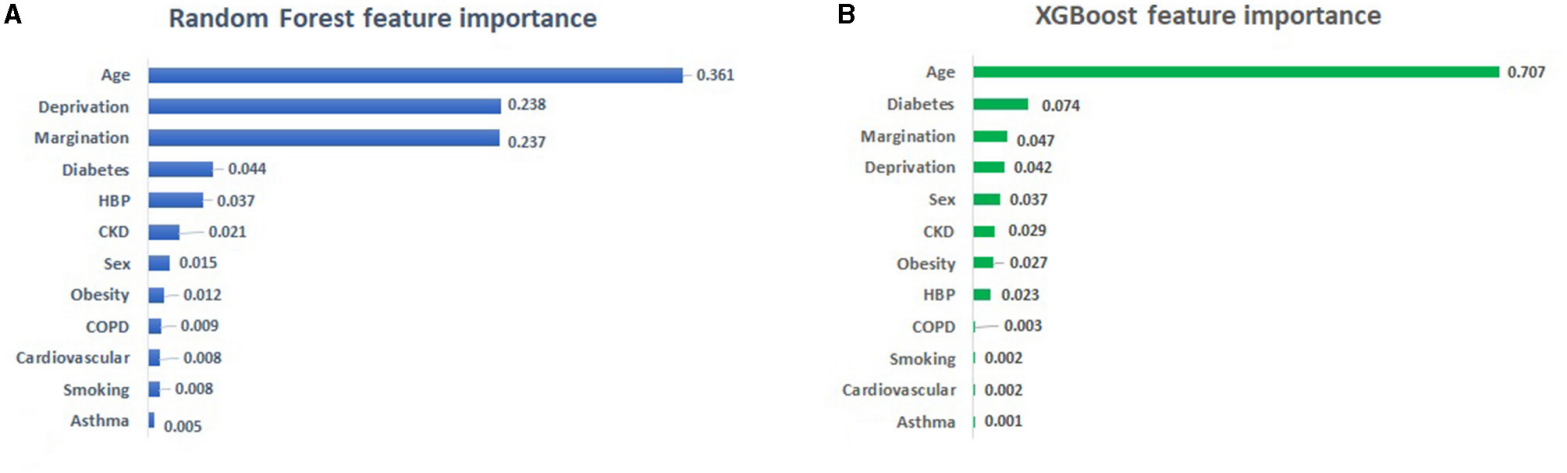
**(A)** Output of RF and **(B)** XGBoost feature importance. HBP, High Blood Pressure; CKD, Chronic Kidney Disease; COPD, Chronic Obstructive Pulmonary Disease.

An RF classifier was implemented using 70% of the dataset to train the algorithm and 30% to test it. An accuracy score of 0.94 was obtained with the RF classifier. The same split of the dataset was used to train and test the XGBoost classifier with an accuracy score of 0.95 (log loss of 1.83). The level of feature importance obtained with RF and XGBoost is shown in Figures 5A, B, respectively.

Figures 5A, B shows that the most important feature obtained by both algorithms is age. According to the feature importance analysis with RF shown in Figure 5A, socioeconomic inequality measured by the deprivation index and the marginalization index are the next features in terms of importance, followed by the prevalence of diabetes and HBP. The feature importance analysis with XGBoost shows that diabetes and social inequalities (deprivation and marginalization) followed by sex are the most important features defining deaths by COVID-19.

## Discussion

This study has shown that economic deprivation and marginalization in Mexico had a great impact on the COVID-19 deaths. As shown in Figure 5, both algorithms confirm age as the main feature defining death by COVID-19, in both cases inequality (marginalization and deprivation) as well as diabetes are in the top five features. The only difference is the fifth feature which is HBP in RF and gender in XGBoost. This difference is explained by the fact that XGBoost measures feature importance based on average gain (XGBoost Documentation, 2023), whereas RF uses total gain as a measure of feature importance (Sckit-Learn Documentation, 2023). In both cases, the importance of a feature is calculated as the total reduction of the criterion brought about by that feature.

**Figure 6:**
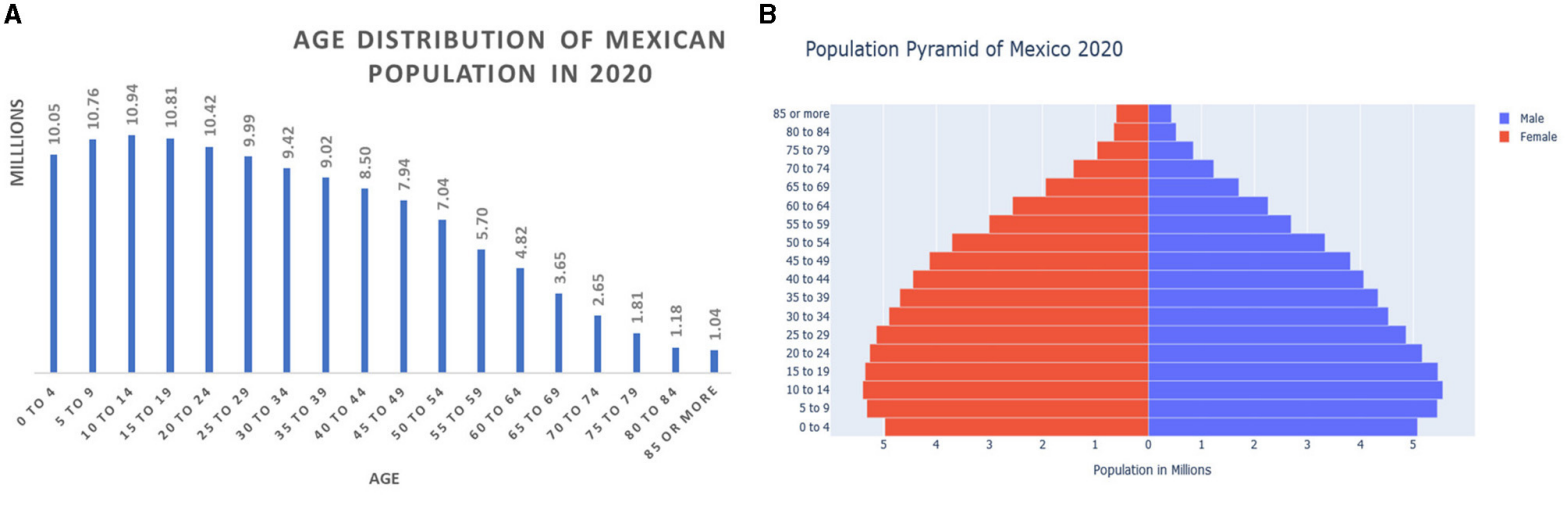
**(A)** Population by age and **(B)** population by sex in Mexico in 2020.

The exploratory data analysis shown in Figure 3 indicates that most of the positive cases were reported by young people (mean age 39 years for both men and women). However, older people, with a mean age of 62 and 64 years of age for men and women, respectively, reported most of the fatal cases of COVID-19. This confirms the results of the feature importance analysis, which identified age as the most important feature. According to census data, the mean age in Mexico in 2020 was 29 years of age, an increase from 22 in 2000, 24 in 2005, 26 in 2010, and 27 in 2015. The population pyramids by age and sex are shown in Figure 6.

At the onset of the pandemic, the first mobility restrictions focused on asking people 65 years of age and older to stay at home and only go out to buy essential items if they could not get someone to buy essential items for them. The same age group was also given priority in the implementation of the vaccination program. Since age is an important factor in determining death from COVID-19, it was correct to implement policies aimed at prioritizing people 65 years of age and older.

The results of the feature importance analysis and the exploratory analysis show that children under 10 years of age were not affected by infection positivity or lethality as much as people over 65 years of age. The low risk of COVID-19 mortality in children and young adults was also found with data from the United States, United Kingdom, Italy, Germany, Spain, France, and South Korea (Bhopal et al., 2021). In addition, in 2020, a few children and young adults in low- and middle-income countries were infected or killed by COVID-19 infection (Zar et al., 2020). In Mexico, a low children mortality rate of ~1.2% of the total of deaths was reported by Stern et al. (2022) through July 2021. The results of this study confirm that although the virus has mutated, children and young adults have not been severely affected by the virus.

The feature importance study shown in Figure 5, defines diabetes as a relevant comorbidity when defining mortality by COVID-19. According to Rojas-Martínez et al. (2018), Mexico is among the top 10 countries in terms of diabetes prevalence, with a prevalence rate of 9.2 % in 2012 (~6.4 million people with diabetes), and it is estimated that diabetes prevalence will reach 12–18% by 2030 (Rojas-Martínez et al., 2018). Health authorities have linked dietary habits to the prevalence of diabetes (ISSSTE, 2019). For example, high consumption of sugar, saturated fat and sodium have been linked to the prevalence of diabetes. In addition, lifestyle factors such as physical inactivity, obesity and genetic factors have also been associated to diabetes (Gobierno de Mexico, 2021). Other studies in other parts of the world have reported diabetes and age as the most important determinants of mortality from COVID-19 (Osibogun et al., 2021; Adab et al., 2022; Al-Otaiby et al., 2022; Usui et al., 2022).

In 2021, diabetes was the third leading cause of death in Mexico and the leading cause of death among adults over the age of 65. It was also the third leading cause of death in men and the second leading cause of death in women in Mexico (INEGI, 2021). As a result, many local governments and health institutions have taken steps to promote a better lifestyle and diet among the Mexican population. Thus, a high mortality rate from COVID-19 would be expected due to the high prevalence of diabetes in Mexico.

Poverty has been associated with an increase in the incidence of type 2 diabetes (Hsu et al., 2012). In addition, unequal access to health services causes inequalities in diabetes treatment and deaths (Ricci-Cabello et al., 2010; Medina-Gómez and Escobedo-De La Peña, 2023). The burden of the COVID-19 pandemic in terms of hospitalizations and critical care added to the strain on Mexico's limited and underfunded public health system, which also cares for diabetes patients.

Using small area estimation techniques and data from the National Health and Nutrition Survey 2018 (ENSANUT, 2018), Mexico's Institute of Geography and Statistics (INEGI) estimated the prevalence of obesity, diabetes, and high blood pressure in all municipalities of the country. The results of this experimental model as well as the details of the model can be found at their website (INEGI, 2023).

The marginalization index dataset was augmented with the estimated percentages previously described in all municipalities. The resulting correlation matrix is displayed in Figure 6.

**Figure 7:**
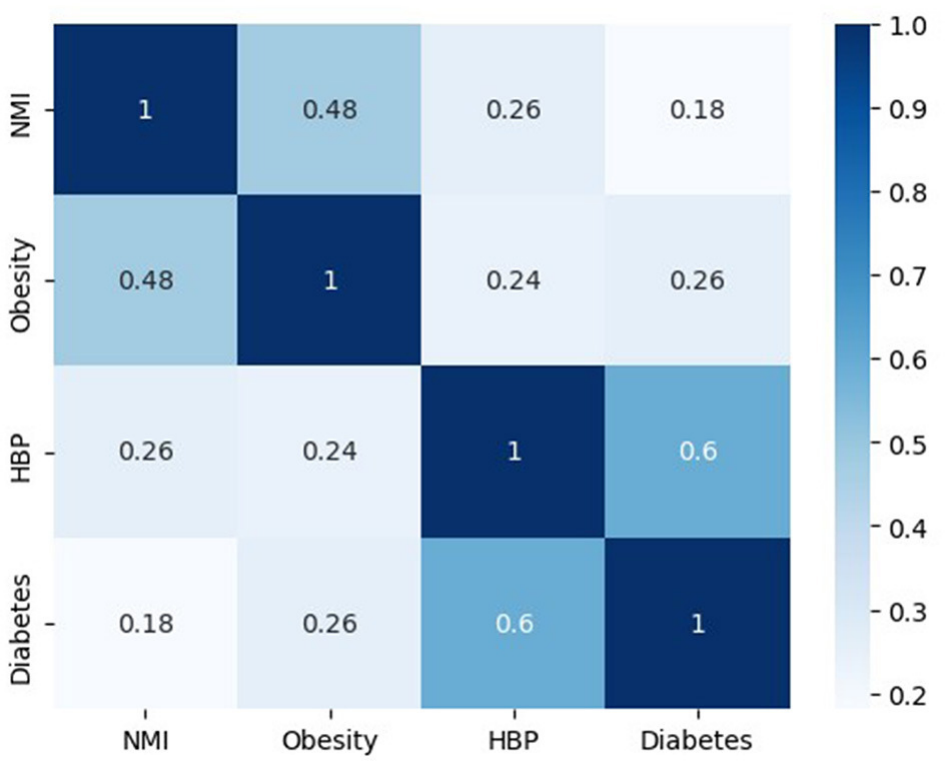
Correlation matrix between the Normalized Marginalization Index (NMI) and estimated comorbidities prevalence in Mexico.

Figure 7 shows that the highest correlations are between diabetes and HBP as well as between marginalization and obesity. The latter correlation has been previously observed in research and it is attributed to food habits, the prevalence of ultraprocessed food (Rojas Martínez, 2022), and food insecurity prevalent in highly marginalized zones (Morales-Ruán et al., 2014; Raccanello, 2020).

The data suggests that there is a correlation between socioeconomic status and an increased incidence of diabetes, as well as a higher mortality rate from COVID-19. Additionally, gender and high blood pressure (HBP) are also significant factors in defining COVID-19 deaths. Figure 3 illustrates that men are more affected than women. Since HBP is associated with diabetes, it is the primary comorbidity affecting the Mexican population and causing deaths from COVID-19 infections. The trained model can predict disease outcomes based on patient age, sex, diabetes prevalence, and the level of marginalization and deprivation in the patient's municipality. For example, a male aged 65 or older with diabetes from a highly marginalized municipality would have a higher probability of developing a severe infection or dying from the infection. The results suggest that public health policies focused on reducing the prevalence of diabetes and obesity in the Mexican population (e.g., improving dietary habits and promoting physical activity) and reducing inequality and deprivation are needed to make the population more resilient to future pandemics. This study complements previous studies in Mexico that used data from the start of the pandemic in 2020 (Kammar-García et al., 2020; Johnson et al., 2021) and adds the effect of inequality in the outcome of the pandemic.

The limitation of this study is that only a few comorbidities were reported in the dataset. Also, all data available in the dataset are self-reported, so comorbidities may not be fully reported, especially in patients in the early stages of the disease without a formal diagnosis. Moreover, the COVID-19 dataset contains residency data at the municipal level therefore marginalization has to be taken at the municipal level which in some cases hides the different levels of marginalization and deprivation within the municipality.

## Conclusion

The data availability on comorbidities of COVID-19 patients and their infection outcomes of their infection enabled the use of machine learning algorithms to study feature importance. Machine learning algorithms are effective tools for analysis Big Data, as demonstrated in this research. Random Forest and XGBoost were the two algorithms used for classification, and they obtained similar feature importance. The two algorithms identified age, gender, diabetes prevalence, and economic inequality as the top 5 features associated with COVID-19 mortality. The analysis presented here indicates that men over 65 experienced the highest number of deaths. Additionally, the study suggests that socioeconomic inequality contributes to the high COVID-19 mortality in Mexico. Therefore, older men with diabetes from marginalized areas are at a higher risk of developing severe COVID-19 infection or dying from it. The analysis suggests that implementing public policies or programs to reduce inequality and the prevalence of diabetes could increase the population's resilience to future pandemics.

## Data availability statement

The original contributions presented in the study are included in the article/supplementary material, further inquiries can be directed to the corresponding author.

## Author contributions

JM-A: Conceptualization, Data curation, Formal analysis, Investigation, Methodology, Project administration, Resources, Software, Supervision, Validation, Visualization, Writing – original draft, Writing – review & editing.
